# Assessment of Correlation between *Androgen Receptor* CAG Repeat Length and Infertility in Infertile Men Living in Khuzestan, Iran 

**DOI:** 10.22074/ijfs.2015.4239

**Published:** 2015-07-27

**Authors:** Saeid Reza Khatami, Hamid Galehdari, Abdorrahman Rasekh, Hayat Mombeini, Elham Konar

**Affiliations:** 1 Department of Genetics, Faculty of Sciences, Shahid Chamran University, Ahvaz, Iran; 2 Department of Statistics, Faculty of Mathematical Sciences, Shahid Chamran University, Ahvaz, Iran; 3 Department of Urology, Golestan Hospital, Ahvaz University of Medical Sciences, Ahvaz, Iran

**Keywords:** Male Infertility, Androgen Receptor, CAG Repeats, Y Chromosome

## Abstract

**Background:**

The *androgen receptor (AR)* gene contains a polymorphic trinucleotide repeat that encodes a polyglutamine tract in its N-terminal transactivation domain (N- TAD). We aimed to find a correlation between the length of this polymorphic tract and azoospermia or oligozoospermia in infertile men living in Khuzestan, Iran.

**Materials and Methods:**

In this case-control study during two years till 2010, we searched for microdeletions in the Y chromosome in 84 infertile male patients with normal karyotype who lived in Khuzestan Province, Southwest of Iran. All cases (n=12) of azoospermia or oligozoospermia resulting from Y chromosome microdele- tions were excluded from our study. The number of CAG repeats in exon 1 of the AR gene was determined in 72 patients with azoospermia or oligozoospermia and in 72 fertile controls, using the polymerase chain reaction (PCR) and polyacrylamide gel electrophoresis.

**Results:**

Microdeletions were detected in 14.3% (n=12) patients suffering severe oligozoospermia. The mean CAG repeat length was 18.99 ± 0.35 (range, 11-26) and 19.96 ± 0.54 (range, 12-25) in infertile males and controls, respectively. Also in the infertile group, the most common allele was 19 (26.38%), while in controls, it was 25 (22.22%).

**Conclusion:**

Y chromosome microdeletions could be one of the main reasons of male infertility living in Khuzestan Province, while there was no correlation between CAG length in *AR* gene with azoospermia or oligozoospermia in infertile men living in Khuzestan, Iran.

## Introduction

It seems that male factor is the reason of infertility in approximately half of infertile cases (1). The most common defects in infertile men are severe oligozoospermia or azoospermia, and these patients frequently undergo assisted reproductive technology (ART), such as intra-cytoplasmic sperm injection (ICSI), which may results in the transmission of these defects to next male generation. Different factors including environmental and genetic factors may cause alternation in sperm production, while several genetic studies have been recently conducted in this regard. The role of androgen as the main male hormone in determination of male sexual differentiation and male secondary sexual characteristics is well known. Also the initiation and maintenance of spermatogenesis is due to well action of this hormone. It is clear that androgen act on target cells with the help of androgen receptor (AR). Locus of the *AR* gene is on X chromosome at position Xq11-12. The *AR* gene with 8 exons can produce AR with three following domains: i. Exon 1 encoding transactivation domain, ii. Exon 2 and 3 encoding DNA-binding domain, and iii. Exon 4-8 encoding C-terminal ligand-binding domain. Exon one has two polymorphic sequences known as CAG and GGN that contain three nucleotide repeats. The CAG repeats encode polyglutamine residues with different length in transactivation domains of the receptors. The CAG repeats are unstable and the number of their repeats may change during meiotic division. Many mutations in *AR* gene cause various degree of androgen resistance (2-4).

A negative correlation between CAG repeats number and AR transcriptional activity has demonstrated *in vitro* system. Some clinical studies have shown association of longer CAG repeat with oligozoospermia and azoospermia (2-7). In contrast, others have not reported this correlation (8- 12). According to these notions, we studied CAG repeats length in *AR* gene in oligozoospermic and azoospermic men living in Khuzestan Province, Southwest of Iran.

## Materials and Methods

### Individuals

In this case-control study during two years till 2010 in Khuzestan Province, Iran, in conformity with the ethical committee, 84 azoospermic or oligozoospermic men were selected according to World Health Organization (WHO) criteria (13) with sperm concentration less than 15 million/ ml as in patient samples. Also, 72 men with normal semen analysis who had at least one child were studied as control samples. Patient samples were collected from Shafa Genetic Lab and the IVF Center of Imam Khomeini Hospital, Ahvaz, Khuzestan Province, Iran, while control samples were obtained from Khuzestan Blood Transfusion Organization, Ahvaz, Khuzestan Province, Iran. Microscopic semen analysis method was used for patient assessment. We selected only patients with idiopathic azoospermia and severe oligozoospermia; therefore, twelve individuals were excluded due to different cases of azoospermia or oligozoospermia resulting from endocrine causes or Y chromosome microdeletions.

An informed consent form and agreement for human specimen analysis were taken from all participants. Peripheral blood samples were stored at -70˚C with ethylenediaminetetraacetic acid (EDTA, Merck, USA) as anticoagulant in order to extract DNA.

### Molecular analysis

Genomic DNA was extracted using Diatom DNA Prep extraction kit (Gene Fanavaran Co., Iran), according to the manufacturer’s instructions. Then 100 μg purified DNA were diluted and stored at 4˚C before analysis. Next using the polymerase chain reaction (PCR, BioRad, USA) for detection of following six sequence-tagged sites (STS): the sY84 and sY86 within the azoospermia factor a (AZFa) region, the sY127 and sY134 in the AZFb area, and the sY254 and sY255 located within the AZFc site on the long arm of the chromosome Y (Table 1). The applied amplification system, recommended by the European Academy of Andrology (EAA), allowed us to detect 90% of the microdeletions in the AZF region (14, 15). The multiplex PCR product was run by electrophoresis on a 3% agarose (Gene Fanavaran Co., Iran) gel impregnated with ethidium bromide at 5 μg/mL concentration and visualized under UV light.

CAG repeats in exon 1 of the *AR* gene were amplified with these following primers: forward primer including 5′-GCT GTG AAG GTT GCT GTT CCT CAT-3′ and reverse primer including 5′-TCC AGA ATC TGT TCC AGA GCG TGC- 3′. Only patients without Yq microdeletions were then analyzed (n=72). We used 25 μl PCR solution (Gene Fanavaran Co., Iran) containing 5 μl PCR buffer, 3 μl of DNA, 0.25 μl of each dNTP, 0.75 μl MgCl_2_, 0.5 U Taq DNA polymerase, and 1 μl of each forward and reverse primer. PCR was done under these conditions: an initial denaturation step at 94˚C for 3 minutes, 40 cycles of denaturation at 94˚C for 15 seconds, annealing at 49˚C for 1 minute and extension at 72˚C for 1 minute. Final step of extension was 10 minutes at 72˚C. To confirm amplification, PCR products were electrophoresed through a 1.5% agarose gel. Then PCR products, were separated on a 8%-polyacrylamide gel with 1X tris-borate-EDTA (TBE, Merck, USA) at 150 V for 8 hours, impregnated with ethidium bromide at 5 μg/ml and visualized under UV light. After comparing all polyacrylamide gels, alleles with the same band and the same size (CAG repeats) were categorized into one group. One allele from each group was selected and sequenced, so the size of the PCR band was determined.

### Statistical analysis

Results are reported as mean ± SD in case and control groups. Statistical analysis was carried out using t test. The data were considered significant when P<0.05.

## Results

In this case-control study, 72 fertile and 84 infertile males were analyzed. Forty-eight patients showed severe oligospermia (sperm concentration ≤15 million/ ml), and 36 were non-obstructive azoospermic males. Summary of clinical data of fertile and infertile groups are shown in table 2. The azoospermic patients aged between 23 and 47 years, with mean age of 31 years. The oligozoospermia patients’ age ranged from 22 to 38 years, with mean age of 32 years. The control group aged between 21 and 67 years, with mean age of 38.79 ± 10.24 years.

**Table 1:** Sequence of primers used to amplify specific regions to assess Y chromosome microdeletions


STS	Region	Sequence 5΄ to 3΄	bp

SRY	Yp11.3	F 5΄-GAA TAT TCC CGC TCT CCG GA- 3΄	472
		R 5΄-GCT GGT GCT CCA TTC TTG AG- 3΄	
Y84	AZFa	5΄-AGA AGG GTC TGA AAG CAG GT- 3΄	325
		5΄-GCC TAC TAC CTG GAG GCT TC- 3΄	
Y86	AZFa	5΄- GTG ACA CAC AGA CTA TGC TTC- 3΄	320
		5΄- ACA CAC AGA GGG ACA ACC CT- 3΄	
Y127	AZFb	F 5΄-GGC TCA CCA ACG AAA AGA AA - 3΄	274
		R 5΄-CTG CAG GCA GTA ATA AGG GA - 3΄	
Y134	AZFb	F 5΄GTC TGC CTC ACC ATA AAA CG- 3΄	301
		R 5΄-ACC ACT GCC AAA ACT TTC AA- 3΄	
Y254	AZFc	F 5΄-GGG TGT TAC CAG AAG GCA AA- 3΄	370
		R 5΄-GAA CCG TAT CTA CCA AAG CAG C- 3΄	
Y255	AZFc	F 5΄-GTT ACA GGA TTC GGC GTG AT- 3΄	126
		R 5΄-CTC GTC ATG TGC AGC CAC- 3΄	


AZF; Azoospermia factor and STS; Sequence-tagged sites.

**Table 2:** Summary of clinical data of fertile and infertile groups


Classification (n)	Age (Y)	Sperm concentration (million/ml)	Bitesticular volume (ml)	Morphology (%)	Motility(%)

Control (72)	21-67 (38.79 ± 10.24)	20-100 (53.19 ± 17.83)	34-70 (49 ± 9.29)	40.08 ± 7.53	60.94 ± 9.89
Total infertile (72)	24-65 (38.62 ± 7.51)	0-4 (0.53 ± 0.62)	2-66 (28.97 ± 14.30)	13.03 ± 9.07	21.56 ± 14.47
Azoospermia (36)	23-47 (31.25 ± 8.05)	0	6-51(24.13 ± 12.73)	0	0
Sever oligospermia (48)	22-38 (32.98 ± 5.21)	0.01-4 (1.09 ± 1.17)	2-60 (25.67 ± 13.61)	11.25 ± 4.78	11.75 ± 4.25


Our results revealed that deletions were found in 12 out of 84 (14.3%) infertile men. Therefore, 1.2% of the infertile men had microdeletions in the AZFa region, 11.9% in the AZFb regions, and 1.2% in the AZFc regions (Table 3). It means that among 48 severe oligozoospermic patients, one had deletions only in the AZFa region, 10 had microdeletions in the AZFb region, and one had in the AZFc region. All patients and controls were shown amplification of the *sex-determining region (SRY)* gene. In control males, no microdeletions were identified (Figes.1, 2).

Then patients with microdeletions were excluded from the CAG repeat analysis (Table 3). After gels analysis (Fig.3), we were able to identify 6 and 12 different alleles in the infertile and control groups, respectively. Due to the facts that males have one X chromosome and individuals belonging to a population have the same allele, we were able to specify the frequency of distribution of the alleles. The distribution of the allele frequencies in both groups is depicted in table 4 and figure 4.

**Table 3:** Frequency of AZF Y chromosome microdeletions in infertile men


Patients	Azoospermia number ( % )	Oligospermia number ( % )	Total number ( % )

Deletions	36 (42.86%)	48 (57.14%)	84 (100%)
AZFa	0	1 (2.1%)	1 (1.2%)
AZFb	0	10 (20.8%)	10 (11.9%)
AZFc	0	1 (2.1%)	1 (1.2%)
Total number ( % )	0	12 (25%)	12 (14.3%)


AZF; Azoospermia factor.

**Fig.1:**
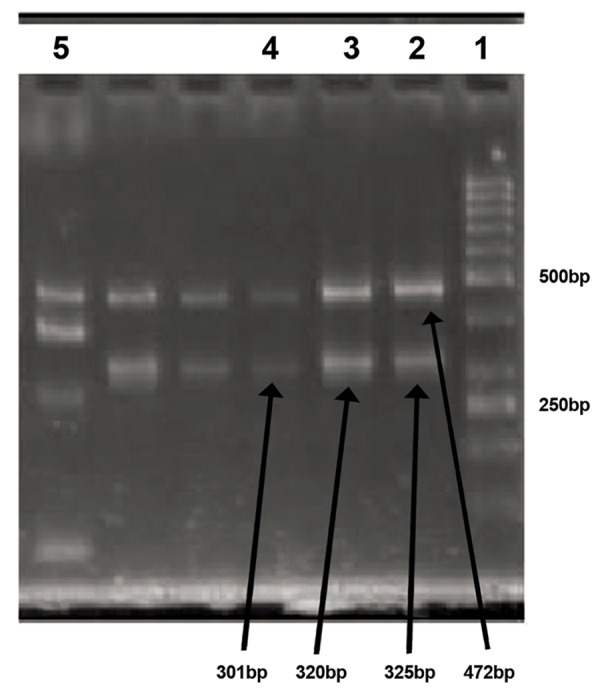
Amplified sequence-tagged sites (STS) in an infertile man with no deletion. line 1; 50 bp marker (two sharp bands are 250 bp and 500 bp markers), line 2; The heavy band is SRY (472bp) and the other is Y84 STS (325bp), line 3; The heavy band is SRY (472bp) and the other is Y86 STS (320bp), line 4; The heavy band is SRY (472bp) and the other is Y134 STS (301bp) and line 5; The heavy band is SRY (472bp) and the others are Y254 (370bp), Y127 (274bp) and Y255 (126bp) STSs.

**Fig.2:**
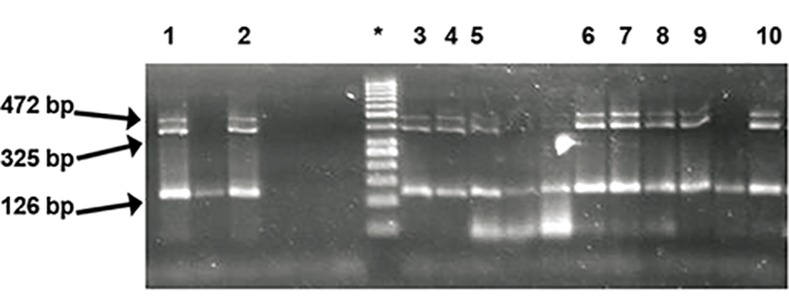
Analysis of polymerase chain reaction (PCR) products using Y chromosome with STS markers on 3% agarose gel indicates 10 infertile men (1-10) with microdeletion in Y127(274 bp). line*; 50 bp marker (two sharp bands are 250 bp and 500 bp markers) and line 1-10; The heavy band is SRY (472bp) and the others are Y84 STS (325bp) and Y255 STS (126bp).

**Fig.3:**
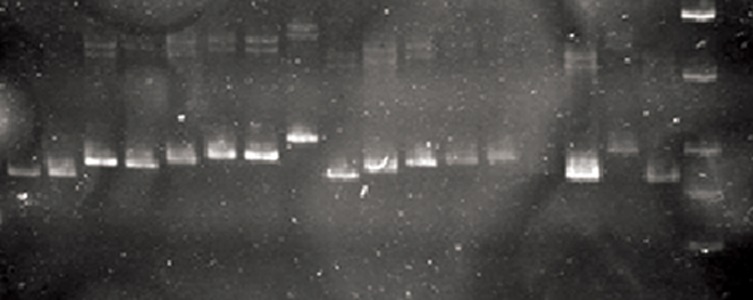
Analysis of polymerase chain reaction (PCR) products using *androgen receptor (AR)* gene on 8% polyacrylamide gel indicates difference between alleles size. First line from right is 10 bp DNA marker that its bands (from down to up) are 10, 20, 30, 40 and 50 bp, while other lines are different CAG alleles.

**Fig.4:**
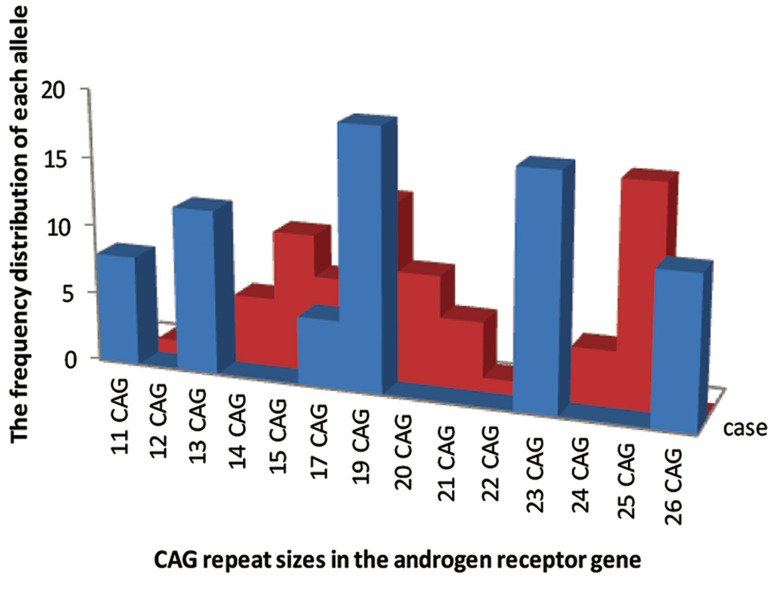
Distribution of CAG repeats sizes in the androgen receptor (AR) gene of infertile men (blue line) and fertile controls (red line).

**Table 4:** Distribution of CAG repeat sizes in the androgen


Group	Allele (the number of CAG )	Number	Frequency

Case	11	8	11.10%
	13	12	16.70%
	17	5	7%
	19	19	26.40%
	23	17	23.60%
	26	11	15.30%
Control	12	1	1.40%
	13	1	1.40%
	14	5	7%
	15	10	13.90%
	17	7	9.70%
	19	13	18.10%
	20	8	11.10%
	21	5	7%
	22	1	1.40%
	23	1	1.40%
	24	4	5.50%
	25	16	22.20%


According to the frequencies, the most and the least common alleles were seen in both groups. In the infertile group, the allele with 19 repeats of CAG was the most common allele (26.38%), while in fertile group, it was the allele with 25 repeats (22.22%). The least allele in case group has 17 repeats, while in the control group, the least alleles were seen with 12, 13, 22 and 23 repeats. The mean CAG repeat length is 18.99 ± 0.35 (range, 11-26) and 19.96 ± 0.54 (range, 12-25) in infertile males and controls, respectively. There is no association between CAG repeat length and azoospermia or oligozoospermia in infertile men living in Khuzestan in this study (Table 5).

**Table 5:** Correlation between severity of impairment of spermatogenesis and CAG length


Group	CAG, mean ± SD	P value	Sperm count	n	CAG, mean ± SD	P value

Infertile	18.99 ± 0.35	NS	Azoospermia	36	19.80 ± 0.75	NS
			Sever oligospermia	36	18.16 ± 0.63	NS
Fertile	19.96 ± 0.54	NS	Normal	72	19.96 ± 0.54	NS


NS; Non significant.

## Discussion

As mentioned, male factor is the reason of infertility in approximately 50% of infertile cases, while the causes of more than half of these cases are poorly understood. It is clear that androgen as the main male hormone has critical roles in male sexual differentiation and regulation of spermatogenesis. It is also noted that androgen can act on target cells through its receptors.

Our study investigated the association between the number of CAG repeats of exon 1 in androgen receptor gene and sperm counts in 72 azoospermic or oligozoospermic men and 72 control men from Khuzestan, Iran. The infertile men with idiopathic azoospermia and severe oligozoospermia as the case group were selected after screening for other reason of infertility such as Yq microdeletion. Among 84 infertile men, 12 individuals (14.3%) with severe oligozoospermia were found to have microdeletions. The frequency of microdeletions was 25% in the severe oligozoospermic group and zero in the azoospermic group. Our findings showed that 1.2% of the infertile men had microdeletions in the AZFa region, 11.9% in the AZFb regions, and 1.2% in the AZFc regions. In other words, among 48 severe oligozoospermic patients, one had deletions only in the AZFa region, 10 had microdeletions in the AZFb region, and one had microdeletions in the AZFc regions. Therefore, 83.3% of deletions were located at the AZFb region.

Although several studies have investigated the proposed association between CAG repeat length in *AR* gene and infertility, these reports have yielded conflicting results. Different studies from the United States, Singapore (2), France (3), Japan (6) and Spain (7) have reported an association between higher CAG repeats and low sperm count, while other studies from Germany (8, 10), India (9), Nigeria (16), Mexico (17), Chile (18) and Egypt (19) have not demonstrated a significant correlation between them. One meta-analysis (20) using 33 published studies understood correlation between CAG repeat number in *AR* gene and infertility in men. The observed variations in the results from previous studies may originate from several factors: i. Ethnically diverse populations which can change some environmental and genetic factors in them, ii. The studied infertile men may represent a heterogeneous group with respect to the causes of infertility and may be under the effect of different genetic mutations or even epigenetic phenomena, and iii. Different inclusion criteria in each study. The infertile populations in different studies may be included various categories of infertility such as patients with varicocele or infection in genital tract and also different semen parameters, for example azoospermia or oligozoospermia (10, 21, 22). Most importantly, the control groups in many of these previous studies were not well-matched in terms of ethnicity and age. The control groups in these studies often included not only individuals with proven fertility, but also individuals with normal sperm count but not proven fertility and/or individuals from unselected populations (23-25).

At first our results showed differences between the cases and controls in the length of CAG repeats. In the case group, it was only found 6 alleles ranging from 11 to 26 repeats of CAG. Diversity between the numbers of CAG repeats in controls is more than the cases, indicating that there are 12 different alleles in fertile individuals ranging between 12 and 25 repeats, while four of the alleles are common in both groups that are alleles with 13, 17, 19 and 23 repeats. Despite that, both groups have differences in the frequencies of the alleles. In group of infertile men, alleles with 13 and 23 repeats have high frequencies, but the numbers of these alleles in control group are very low.

Also in fertile men, we did not find alleles with repeat number of 11 and 26, but these alleles showed high frequencies in infertile men. It can be mention that in case group, the allele with 19 CAG repeats is the most common, but in control group, the most common allele has 26 repeats.

Despite the facts we found no differences in the mean number of CAG repeats between infertile men (18.99 ± 0.35, range, 11-26) and controls (19.96 ± 0.54, range, 12-25). The infertile group was further subdivided according to sperm counts, and no differences were found in any subgroup when compared to controls. These results are in agreement with studies in which no association was found. Thus, it might be assumed, at least in our studied population, the AR could act without being associated with any pathologic phenotype.

Our study presented the range of alleles in an ethnically and geographically restricted population of Iranian men with normal fertility. There was no significant correlation between CAG repeat length and risk of male infertility in our ethnically restricted experimental population compared with the matched control population. In other word, polymorphism detected in the CAGrich region of the *AR* gene may not be a useful genetic indication of male factor infertility.

## Conclusion

With this high percentage of deletion in Yq chromosome in oligospermic men in this study, we can say that Y microdeletions is likely to be one of the main reasons of male infertility in this region, and the most frequent type of microdeletion is located at the AZFb region. Also, in this study, we did not find a correlation between CAG repeats in *AR* gene and sperm count, showing that there was no significant correlation between CAG repeat length and the risk of male infertility in this part of Iran. Therefore, polymorphism detected in the polyglutamine-rich region of the AR could not be a useful genetic indication of male infertility.
